# Hybrid Antibody–Aptamer Assay for Detection of Tetrodotoxin in Pufferfish

**DOI:** 10.1021/acs.analchem.1c03671

**Published:** 2021-10-26

**Authors:** Xhensila Shkembi, Vasso Skouridou, Marketa Svobodova, Sandra Leonardo, Abdulaziz S. Bashammakh, Abdulrahman O. Alyoubi, Mònica Campàs, Ciara K. O′Sullivan

**Affiliations:** † Interfibio, Nanobiotechnology and Bioanalysis Group, Departament d’Enginyeria Química, Universitat Rovira i Virgili, Avinguda Paisos Catalans 26, 43007 Tarragona, Spain; ‡ IRTA, Ctra. Poble Nou km 5.5, 43540 Sant Carles de la Ràpita, Spain; § Department of Chemistry, Faculty of Science, King Abdulaziz University, P.O. Box 80203, 21589 Jeddah, Kingdom of Saudi Arabia; ∥ Institució Catalana de Recerca I Estudis Avancats (ICREA), Passeig Lluís Companys 23, 08010 Barcelona, Spain

## Abstract

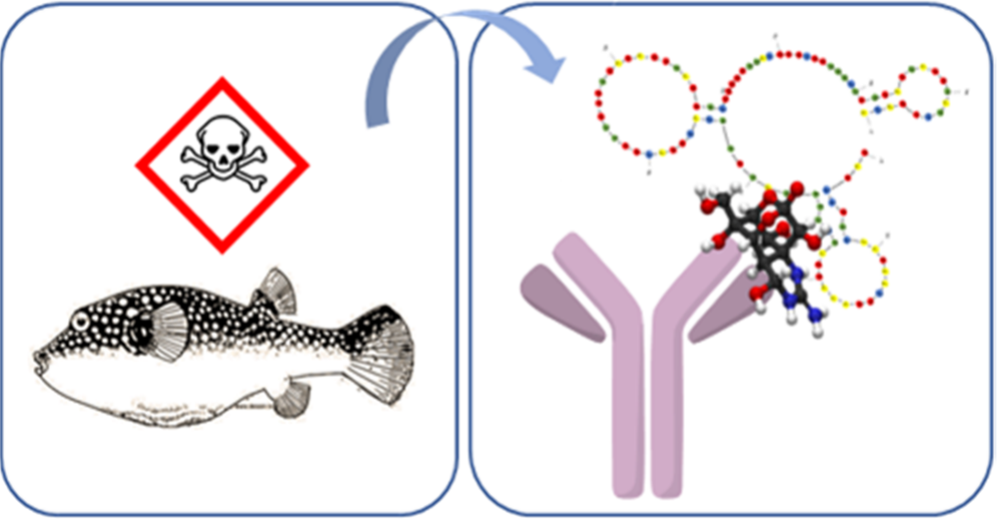

The marine toxin tetrodotoxin (TTX) poses a great risk to public health safety due to its severe paralytic effects after ingestion. Seafood poisoning caused by the consumption of contaminated marine species like pufferfish due to its expansion to nonendemic areas has increased the need for fast and reliable detection of the toxin to effectively implement prevention strategies. Liquid chromatography-mass spectrometry is considered the most accurate method, although competitive immunoassays have also been reported. In this work, we sought to develop an aptamer-based assay for the rapid, sensitive, and cost-effective detection of TTX in pufferfish. Using capture-SELEX combined with next-generation sequencing, aptamers were identified, and their binding properties were evaluated. Finally, a highly sensitive and user-friendly hybrid antibody–aptamer sandwich assay was developed with superior performance compared to several assays reported in the literature and commercial immunoassay kits. The assay was successfully applied to the quantification of TTX in pufferfish extracts, and the results obtained correlated very well with a competitive magnetic bead-based immunoassay performed in parallel for comparison. This is one of the very few works reported in the literature of such hybrid assays for small-molecule analytes whose compatibility with field samples is also demonstrated.

## Introduction

Tetrodotoxin (TTX) is a very potent neurotoxin produced by marine bacteria, and it is associated with severe seafood poisoning after consumption of pufferfish (Tetraodontidae family).^1^ Its paralytic toxic effects derive from its selective binding to voltage-gated sodium channels and ultimately interfering with the neural transmission.^2^ Symptoms of TTX intoxication include numbness sensation in the mouth, headache, vomiting, and muscle weakness,^3^ and fatal respiratory or heart failure have also been reported.^4^ This low-molecular-weight toxin (319.3 g/mol) was originally isolated from pufferfish in 1909^5,6^ and was later also found in other marine^7^ and terrestrial^8^ species. Even though it was initially believed that TTX was produced by the pufferfish itself, marine bacterial species have been postulated to be able to produce TTX,^9^ suggesting that symbiotic marine bacteria could be the primary source of TTX that bioaccumulates in pufferfish and other marine species and finally reaches humans through the food chain. As recently reported, there are more than 30 different bacteria genera capable of producing TTX that have been isolated, among which the most common is the *Vibrio* sp.^10^ To date, however, there is still some discussion regarding the TTX production/biosynthesis as well as the pathway of TTX bioaccumulation in marine ecosystems.^11^

Pufferfish poisoning is typical of warm waters and was regarded as a problem confined to Asian countries,^1,12^ including Thailand,^5^ Taiwan,^13^ Singapore,^14^ Cambodia,^15^ Bangladesh,^16^ and India.^17,18^ However, toxic pufferfish species have expanded to other regions, and there have been an increasing number of reports of incidences in the Mediterranean Sea, which has been attributed to the opening of the Suez Canal (the “Lessepsian migration”), which resulted in the migration of species from the Red Sea to colonize the Mediterranean Sea,^19−22^ the Aegean Sea,^23^ the Adriatic Sea,^24^ and Oman,^25^ and there have also been reports of the incidence of tetrodotoxin in Australia^26^ and the United States,^27^ highlighting the widespread distribution of the toxin.

Additionally, TTX has been recently found in shellfish, particularly in European countries such as the United Kingdom,^28^ Portugal,^29,30^ Greece,^31^ the Netherlands,^32^ Spain,^33^ Italy,^34^ and France,^35^ although usually at very low concentrations. Nevertheless, it is now considered that TTX may pose a food safety risk even in nonendemic areas.

TTX is highly toxic. Pufferfish poisonings have revealed that ingestion of 0.18–0.2 mg of TTX might be near the minimum dose for developing TTX symptoms, and 2 mg is a lethal dose. However, the levels of TTX that result in acute toxicity or death in humans are still unclear, with some reports of human cases suggesting that acute poisoning can occur from doses of 4–42 mg/kg body weight or higher.^36^ In Japan, where pufferfish is considered a delicacy and highly consumed despite its potential toxicity, a limit of 2 mg of TTX equiv/kg has been used as a criterion to judge the acceptability of pufferfish as food,^37^ and a guide with the edible parts and species of pufferfish that are allowed for consumption has been published.^38^ In the USA, strong restrictions exist for the importation of pufferfish.^39^ In Europe, fish of the family Tetraodontidae and products derived from them must not be placed on the markets.^40,41^ Regarding shellfish, no regulations exist. Nevertheless, the European Food Safety Authority (EFSA) has recently published that concentrations below 44 μg of TTX equiv/kg shellfish meat do not result in adverse effects in humans.^36^

There are about 30 TTX analogues.^42^ Toxicity equivalency factors (TEFs) for these TTX analogues are essential for the evaluation of relative risk, but, unfortunately, information on the relative potencies of TTXs is limited. Although the use of different cell lines in toxicity assays has been questioned, it is evident that most analogues are much less toxic than TTX^43,44^ Additionally, the parent TTX is usually the most abundant.^36^

Bioassays, instrumental analysis, and immunological methods are typically employed to detect TTXs in field samples, based on the toxic effects, physicochemical properties, and antigenic specificity of the toxin, respectively.^45^ Ethical concerns and low specificity of the mouse bioassay, the most frequently used method, encouraged the development of alternative strategies. Liquid chromatography coupled with mass spectrometry (LC-MS/MS)^30,31^ has been widely exploited for TTX detection, and it provides more sensitive, specific, and accurate results than bioassays. However, instrumental analysis techniques are expensive, time-consuming, and labor-intensive and require sample pretreatment, trained personnel, and significant laboratory infrastructure. Immunological methods such as ELISA using specific TTX antibodies can provide quantitative and sensitive detection,^46,47^ and commercial ELISA kits are readily available. The small size of TTX requires the use of hapten-carrier protein bioconjugates for antibody development, requiring careful consideration in the preparation of these bioconjugates. The development of antibody pairs for sandwich assay development is hindered by the small size of the TTX, thus requiring the design of competitive assays.^48,49^ Competitive immunoassays are more difficult to optimize, and the preparation of toxin-reporter molecule conjugates required for some types of immunoassays can also be challenging.^45^ Nevertheless, antibody-based biosensors have been reported and are particularly useful for rapid screening purposes.^50−52^

Aptamers are biorecognition molecules considered as an alternative to antibodies that are suitable for the detection of virtually any type of target.^53−55^ Aptamers are single-stranded synthetic oligonucleotides that can bind their target molecule with high affinity and specificity owing to the specific structural conformations they adopt. Systematic evolution of ligands by exponential enrichment, commonly known as SELEX, was developed for the generation of aptamers, and it is based on iterative rounds of binding, partitioning, and amplification.^56,57^ Compared to antibodies, aptamers show several advantages for biosensing applications, including *in vitro* selection, the possibility to bind any kind of target, high affinity and specificity, reproducible chemical synthesis, stability at various environmental conditions, reversible denaturation, and easy site-directed modification.^53,55^

The development of aptamers for small molecules is a challenging task.^54,58^ One of the main hurdles is target immobilization on a solid matrix to allow selection through traditional SELEX approaches. Altering the native structure of the target to facilitate immobilization can prevent the aptamer from binding to the target in solution in its natural form, and an absence of functional groups can completely hinder immobilization as the small size of the targets also limits the availability of binding sites. An alternative selection strategy, termed *capture-SELEX*, based on library immobilization and use of the target in solution, was first reported by Stoltenburg et al.^59^ This approach is ideal for small molecules since the target molecule can be used in solution, and the potential structure-switching properties of the selected aptamers can be exploited for characterization and assay development.^54,58^ Capture-SELEX strategy has been successfully used for several targets, including aminoglycoside antibiotics,^59^ cadmium,^60^ penicillin,^61^ quinolone,^62^ and lipopolysaccharides.^63^

The path from aptamer discovery to assay development for small molecules is not trivial. The usual format is competitive assays, which can be difficult to develop as discussed earlier in the case of antibodies. Sandwich assays are hindered by the small size of the targets, and to the best of our knowledge, no sandwich aptamer assays have been reported for small molecules. Alternatively, split aptamers can be generated and have been exploited in a sandwich format for the detection of small molecules.^64^ However, the trial-and-error nature of the process of generating split aptamers, possibly resulting in lower binding affinities of the individual fragments and further requirements for modifications, is among the factors discouraging researchers from undertaking this complex and costly task. In fact, to date, split aptamers have only been reported for 15 small molecules.^64^ Hybrid antibody–aptamer sandwich systems, on the other hand, have emerged as an attractive alternative offering the best of both antibody and aptamer biorecognition molecules, together with the advantages of sandwich assays.^65^ Even though several examples have been reported for the detection of protein targets using such hybrid systems, only a handful of examples exist for small molecules, including trinitrotoluene,^66^ tetracycline,^67^ and aflatoxin B1.^68^

Two TTX aptamers have been reported, the first one by Shao et al.,^69^ who did not provide details regarding the selection process or the aptamer affinity, and the second by Gu et al.,^70^ who used a variation of the capture-SELEX strategy with magnetic reduced graphene oxide to immobilize the ssDNA library and identified a TTX aptamer with high affinity (*K*_D_ of 44 nM). In this work, we sought to apply the capture-SELEX strategy to develop TTX-binding aptamers and apply them for the detection of the toxin in pufferfish. Two selections were performed in parallel, using two different types of streptavidin magnetic beads to facilitate library immobilization. Next-generation sequencing of various pools from the selections enabled the identification of aptamer candidates, and different approaches were used to evaluate their binding properties. Finally, a highly sensitive hybrid antibody–aptamer sandwich assay was developed and successfully exploited for the detection of TTX in pufferfish.

## Experimental Section

### Materials

Tetrodotoxin of 98.8% purity (TTX) was purchased from Tocris Bioscience (Bristol, UK) and Latoxan (Valence, France), and standard solutions at 1 mg/mL were prepared in 0.1 M sodium acetate buffer pH 4.8. Certified reference materials of saxitoxin (STX) and domoic acid (DA) were obtained from the National Research Council of Canada (NRC, Halifax, Canada). The mouse monoclonal anti-TTX antibody (CABT-L3089, CD Creative Diagnostics) was obtained from Deltaclon S.L. (Spain). Sulfo-NHS-acetate, maleimide-activated microplate strip wells, Dynabeads M-270 streptavidin magnetic beads (Dynabeads SA-MB; 10 mg/mL, 2.8 μm diameter, 200 pmol biotinylated oligonucleotide/mg particles binding capacity), DreamTaq DNA polymerase, and lambda exonuclease were purchased from Fisher Scientific (Spain). The DNA purification kits (Oligo Clean & Concentrator kit and DNA Clean & Concentrator kit) were purchased from Ecogen (Spain). Okadaic acid potassium salt (OA) from *Prorocentrum concavum*, 11-amino-1-undecanethiol hydrochloride (MUAM), cysteamine, l-arginine, 1,6-anhydro-β-d-mannopyranose, and streptavidin-horseradish peroxidase (SA-HRP) were purchased from Merck (Spain). Maleimide-activated magnetic beads (30 μm diameter, protein binding capacity ≥ 15 mg/mL) were purchased from Cube Biotech (Germany) and SiMAG-streptavidin magnetic beads (SiMAG SA-MB; 10 mg/mL, 1 μm diameter, 80–200 pmol biotinylated oligonucleotide/mg particles binding capacity) from Chemicell (Germany). Streptavidin-polyHRP80 (SA-pHRP) was purchased from Bionova (Spain) and the TMB Super Sensitive One Component HRP Microwell Substrate from Surmodics. All oligonucleotides were synthesized by Biomers.net (Germany).

### Capture-SELEX Process

The library used for the selection was based on a previous report (5′-ATACCA GCTTAT TCAATT-N10-TGAGGC TCGATC-N40-AGATAG TAAGTG CAATCT-3′).^34^ The docking site (5′-TGAGGCTCGATC-3′, 12 nucleotides) was flanked by two random regions of 10 and 40 nucleotides. Library immobilization on streptavidin magnetic beads (SA-MB) was achieved via hybridization of a docking probe (5′-biotin-TEG-GTC-HEGL-GATCGAGCCTCA-3′, where TEG and HEGL are triethylene glycol and hexanethylene glycol spacers, respectively) with the docking site of the library. Two different types of SA-MB beads were used for two parallel selections, the Dynabeads M-270 streptavidin and the SiMAG-streptavidin. The binding buffer used was PBS with 1.5 mM MgCl_2_. A total of 23 selection rounds were performed using the TTX precursors l-arginine and 1,6-anhydro-β-d-mannopyranose as counter-selection molecules during the last six rounds. A detailed description of the selections performed can be found in the Supporting Information.

### Next-Generation Sequencing (NGS) and Data Analysis

Different rounds from the selections were chosen for NGS. Target elution fractions from rounds 6, 9, 16, 23, and counter elution fraction from round 23 for both selections were individually amplified with different forward primers (containing distinct barcode sequences) and a common reverse primer. The resulting dsDNA for each round was column-purified and sequenced using Ion Torrent NGS. The FASTQ raw data were imported into the Galaxy web server (https://usegalaxy.org/), and the quality of the data was evaluated with the “FASTQC” tool, which also provided general statistics. The format of the data was converted to FASTA, and datasets containing only library-length sequences (90–110 bp) were created. Each dataset was finally collapsed to identify unique sequences within the first megabyte of data. The 100 most abundant sequences from all of the datasets were compared to identify the ones preferentially enriched in the target pools. Multiple sequence alignments were performed with Clustal Omega (https://www.ebi.ac.uk/Tools/msa/clustalo/) to determine sequence families, while sequence motif analysis was performed using MEME (https://meme-suite.org/meme/tools/meme). Ten aptamer candidates were finally selected, five from each selection, for further characterization.

### Determination of Affinity Dissociation Constants (*K*_D_)

#### Apta-PCR Affinity Assay (APAA)

The APAA was carried out as previously described with minor modifications specific to TTX.^71^ The APAA was performed using TTX immobilized on maleimide-activated magnetic beads (TTX-beads) in combination with unmodified aptamer sequences. The preparation of the TTX-beads is described in the Supporting Information. For the binding studies, 50 μL of different concentrations of each aptamer (up to 600 nM in binding buffer) was incubated with 2 μL of the TTX-beads for 30 min under rotation at an ambient temperature. The supernatants were discarded, the beads were washed three times with 100 μL of PBS with 0.05% v/v Tween-20 (PBST), and finally resuspended with 20 μL of binding buffer. Bound sequences were detected after PCR amplification using library-specific primers and agarose gel electrophoresis. Analysis was performed in duplicate for each concentration. The intensity of the DNA bands was estimated with ImageJ software and the gel analysis option, plotted against aptamer concentration using GraphPad Prism 6 software, and the *K*_D_ of each aptamer was finally determined using the “One site Specific binding with Hill slope” model.

#### Bead-Enzyme-Linked Aptamer Assay (Bead-ELAA)

The APAA was carried out as previously described with minor modifications specific to TTX.^72^ TTX-beads were used in combination with 5′-biotin-modified aptamers. TTX-beads (2 μL) were mixed with different concentrations of each biotinylated aptamer in binding buffer (50 μL of up to 450 nM) and incubated for 30 min at an ambient temperature under rotation. The supernatants were discarded, and the beads were washed three times with 100 μL of PBST. Next, 50 μL of 50 ng/mL SA-pHRP in PBST was added and incubated for 20 min. After a final washing step (five times with 100 μL of PBST), 50 μL of the TMB substrate was added, and following a brief incubation at room temperature, an equal volume of 1 M H_2_SO_4_ was added to stop color development. The supernatants were separated from the beads using a magnet, transferred to a 96-well microtiter plate, and the absorbance was recorded at 450 nm. The *K*_D_ of the aptamers was calculated as described above. All measurements were carried out in duplicate.

### Hybrid Antibody–Aptamer Sandwich Assay for Determination of TTX

A sandwich assay was developed using an antibody for capture and an aptamer for detection of TTX. Specifically, 50 μL of 5 μg/mL anti-TTX monoclonal antibody in 50 mM carbonate buffer pH 9.4 was used to coat the wells of a MaxiSorp immunoassay plate overnight at 4 °C. The wells were washed three times with 200 μL of PBST, followed by blocking with 200 μL of 1% w/v BSA in PBST for 30 min. The wells were washed again and incubated with 50 μL of different concentrations of TTX in PBS for 1 h. After washing, 50 μL of 500 nM biotinylated aptamer in binding buffer was added and left to incubate for 1 h, followed by washing. Fifty microliters of 100 ng/mL SA-HRP in PBST was then added, followed by a final incubation of 30 min and washing. The TMB substrate (50 μL) was added, and color development proceeded for 6 min. Sulfuric acid (50 μL of 1 M) was added to stop color development, and the absorbance was recorded at 450 nm. All incubation steps were performed at an ambient temperature (22–25 °C) unless stated otherwise. All five aptamer candidates were initially screened in combination with the antibody at a constant TTX concentration (32 μg/mL = 100 μM), and the aptamer providing the highest signal was chosen for the final assay. A calibration curve was constructed using serial twofold dilutions of TTX in the range of 0.039–40 ng/mL (0.12–125 nM). Duplicate measurements were performed, and the data were fitted to a four-parameter sigmoidal model using GraphPad Prism 6 software. The limit of detection (LOD) was interpolated from the curve as the bottom of the fitted curve plus three times its standard deviation (bottom + 3 × SD_bottom_). The precision of the assay was evaluated using duplicate measurements of different concentrations of TTX analyzed on four different days. The interassay coefficients of variation (% CV) were calculated as the standard deviation for each measurement divided by the average. The cross-reactivity of the assay with possibly interfering marine toxins such as domoic acid, okadaic acid, and saxitoxin was finally studied under the conditions detailed above using each toxin at 40 ng/mL.

### Fish Samples and TTX Extraction

Fish extracts were obtained from previous work.^73^ One oceanic pufferfish (*Lagocephalus lagocephalus*, Linnaeus, 1758) (TTX-free individual) and one silver-cheeked toadfish (*Lagocephalus sceleratus*, Gmelin, 1789) (TTX-containing individual) were caught in 2014 in Alicante (Spain). Pufferfishes were dissected, and the gonads, liver, skin, and muscle were retrieved. A double TTX extraction was performed with 0.1% v/v acetic acid, as previously described.^73^ Extracts were obtained at a tissue concentration of 200 mg equiv/mL.

### Detection of TTX in Pufferfish

The compatibility of the hybrid sandwich assay with field sample analysis was initially evaluated with a spiking experiment. The TTX-free extracts from the *L. lagocephalus* pufferfish organs (gonads, liver, skin, and muscle) were spiked with TTX at 1.5 ng/mL, and recoveries were calculated after interpolation in the TTX calibration curve constructed in PBS buffer as detailed above. The *L. sceleratus* TTX-containing fish extracts were then analyzed. The amount of TTX in these extracts was calculated after interpolation in the calibration curve constructed in PBS and also in calibration curves constructed in parallel using the respective extracts from the TTX-free *L. lagocephalus* pufferfish. The extracts were diluted 1/1000 with PBS for all experiments. For comparison, the extracts were also analyzed with a magnetic bead-based competitive immunoassay, as detailed in the Supporting Information.

## Results and Discussion

### Selections

**Figure 1:**
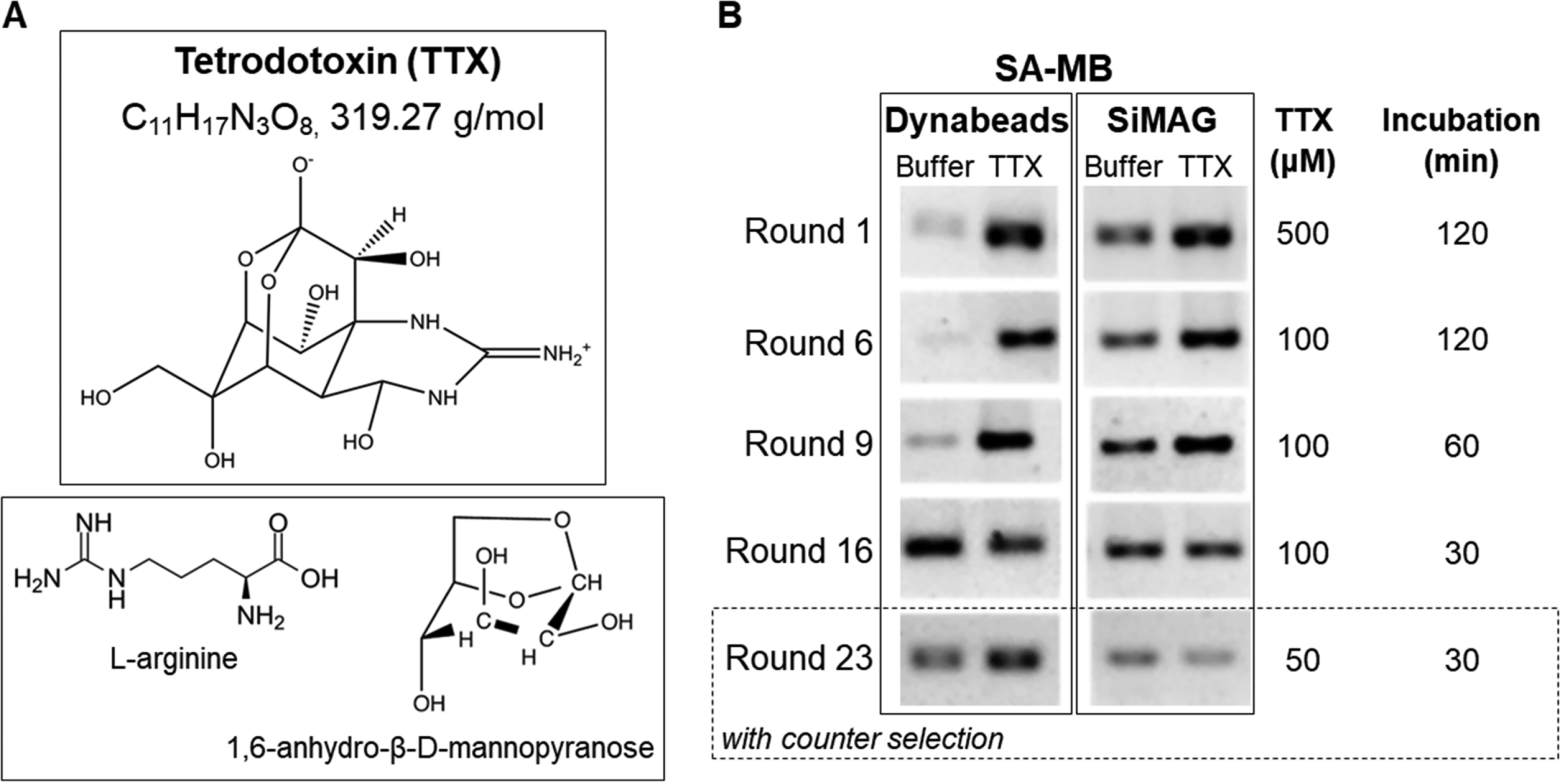
Selection of TTX-binding aptamers. (A) Structures of the target TTX (upper panel) and the counter-selection molecules (lower panel). (B) Evolution of the selections using Dynabeads and SiMAG SA-MB. DNA eluting in the presence of buffer alone or TTX under the specific conditions from the selected rounds was detected after PCR amplification.

TTX is a very small molecule with only one (amine) functional group (Figure 1A). Its covalent linking to a solid matrix to facilitate the partitioning of bound from unbound sequences with traditional aptamer selection approaches would significantly alter its structure and possibly complicate the recognition of the native molecule by the aptamers. Capture-SELEX was thus considered as the most appropriate selection strategy using the ssDNA library immobilized on magnetic beads and the target in solution, rendering the whole molecule accessible for aptamer binding. The design of the ssDNA library was based on a previous report.^59^ Besides the primer annealing sites, the library contained two random regions separated by a docking sequence, which provided an immobilization site to streptavidin magnetic beads through its hybridization with a complementary biotinylated docking probe. Previous studies exploiting the capture-SELEX strategy reported the use of Dynabeads M-270 SA-MB^61,62^ and the library design from the original study.^59^ Different affinity media like streptavidin agarose beads and homemade avidin-magnetic beads were reported in other studies, in combination with libraries containing only one random region whose immobilization was achieved via a biotinylated complementary to one of the primer annealing sites.^60,63^ The distribution of the random sequences on the SA-MB, which could be partially determined by the availability of immobilization sites on the beads and the specific three-dimensional structures of the sequences, could potentially affect the evolution of a selection based on the capture-SELEX strategy. In this work, two different types of streptavidin magnetic beads were used to perform two parallel selections. Even though Dynabeads and SiMAG SA-MB differ in size (2.8 and 1 μm, respectively), their maximum binding capacity is almost identical. Taking into consideration the higher cost of the Dynabeads SA-MB as compared to the SiMAG ones, selections with both bead types were performed in an effort to reduce the overall selection costs and investigate the effect of the properties of the beads on their performance for capture-SELEX applications.

Two selections were performed using the conditions summarized in Table S1 (SI). Starting with 500 μM TTX and 2 h incubation steps (background and target elution steps), the gradual decrease of the TTX concentration and duration of the incubation steps led to the completion of the selections after 23 rounds using 50 μM TTX and 30 min incubations. TTX precursors l-arginine and 1,6-anhydro-β-d-mannopyranose^74^ were added as counter-selection molecules during the last seven selection rounds to improve the specificity of the selected sequences (Figure 1A). The evolution of the selections was monitored by PCR amplification of the background and target elution fractions (Figure 1B). Interestingly, when Dynabeads SA-MB were used, few sequences eluted in the presence of buffer alone, resulting in lower intensity bands after PCR amplification, as opposed to SiMAG beads. This could be a consequence of a better distribution of the docking probe on the larger surface of the Dynabeads facilitating a more efficient hybridization of the random sequences. By the end of the selections, where both the TTX concentration and incubation times were decreased 10-fold and fourfold, respectively, as compared to the initial conditions, the pool from the Dynabeads selection appeared to be more enriched in TTX-specific sequences than the SiMAG one.

### NGS and Identification of Aptamer Candidates

High-throughput sequence analysis of multiple rounds from each selection was performed using Ion Torrent NGS. Five rounds were chosen from each selection, and these were rounds 6, 9, 16, 23, and 23-counter (Figure 1B). Rounds 6 and 16 were chosen because they were performed before a significant change in selection conditions, such as duration of incubation steps (2 h in round 6 → 1 h in round 7) or the concentration of TTX (100 μM in round 16 → 50 μM in round 17). Additionally, in round 9, a significant enrichment in target-eluting sequences was observed by pilot PCR, especially when Dynabeads SA-MB were employed. Finally, round 23 was chosen as the last selection round. A comprehensive bioinformatics analysis was carried out using various tools from the Galaxy web server and other servers, as detailed in the Experimental Section. General statistics can be found in Table 1.

**Table 1:** NGS Data Analysis of Selected Pools from the Two Selections

selection round	total sequences	% GC	sequences 90–110 bp	% unique sequences
(a) Selection with Dynabeads SA-MB				
D6	43 188	42	41 225	99.5
D9	228 862	42	206 990	98.0
D16	82 059	43	76 140	78.2
D23	32 789	41	31 207	62.8
D23-counter	36 727	41	34 937	60.8
(b) Selection with SiMAG SA-MB				
C6	46 902	43	45 334	99.4
C9	54 139	42	50 880	99.2
C16	72 199	42	68 414	95.8
C23	81 705	41	58 299	73.7
C23-counter	111 076	41	76 770	71.5

Enrichment was observed by the end of both selections. The pools in round 6 were highly diverse, containing more than 99% of unique sequences. By the end of round 23, however, the percentage of unique sequences decreased to 62.8 and 73.7% for the Dynabeads and SiMAG SA-MB selections, respectively. Furthermore, the enrichment of the counter-selection pools from the last round for both selections was very similar to the respective target pools from the same rounds. Interestingly, faster enrichment was achieved when Dynabeads SA-MB were used, as by round 16, the percentage of unique sequences dropped to 78.2%, whereas it was 95.8% for selection with the SiMAG beads. Favorable orientation and spacing between sequences on the Dynabeads SA-MB could potentially contribute to faster evolution.

Comparison of the composition of the target and counter-selection pools in rounds 23 revealed the presence of most of the sequences in both datasets. This finding was not surprising since the counter-selection molecules used were structurally almost identical to parts of the TTX molecule. Nevertheless, it was considered that sequences with lower affinity binding to small parts of the target structure could be eliminated during the successive rounds of counter selection/target selection. The evolution of the 20 most enriched sequences (highest counts per million, CPM) in the target pool datasets from rounds 23 was monitored, and their distribution in the pools from rounds 16, 23, and 23-counter is shown in Table S2. A few sequences appeared to have been selectively enriched in the TTX pools as compared to the counter-selection pools, and these were included in the analysis. Rounds 6 and 9 were excluded since low enrichment was observed.

A 7–89-fold enrichment was observed for the sequences selected with Dynabeads SA-MB, which was calculated as the ratio of abundance in round 23 to round 16. The selection performed with the SiMAG beads exhibited 2–73-fold enrichment. This data again demonstrates that the Dynabead-based selection appears to be more successful with a higher enrichment of selected sequences. A direct comparison of the datasets from the last selection rounds with TTX and the counter-selection molecules revealed that the top 20 sequences were slightly more abundant in the counter-selection dataset than in the target dataset when Dynabeads were used (Figure S1). The opposite was observed for the SiMAG-based selection (Figure S2). Notably, sequences selected with one type of beads were not found in the pools from the selection conducted with the other type of beads. Despite theoretically starting from the same initial library and using the same selection conditions, each of the SELEX evolved differently, resulting in different sequences being selected, depending on the beads used for library immobilization. This can be explained, in part, to be due to the fact that even though the starting aliquots are taken from the same initial library, each aliquot can contain a different combination of diverse sequences. Additionally, the size and nature of the beads can affect the number of docking probes and thus the individual sequences of the immobilized library captured on its surface, and this can affect the accessibility of the target to the individual sequences.

Multiple sequence alignment of the 100 most abundant sequences in rounds 23 from both selections was also performed to identify possible sequence families. As can be seen in Figure S3 for the selection carried out with the Dynabeads, only one major cluster was observed, and it contained the most abundant sequence in this dataset, identified as sequence 1, which constitutes 2.1% of the total unique sequences (Table S2). The second and third most abundant sequences, identified as sequences 2 and 3, were encountered at lower percentages (1.1 and 0.9%, respectively) and did not appear to belong to any family. Only one major sequence family was also observed in the dataset from the SiMAG beads selection (Figure S4), containing the second most abundant sequence (sequence 2 at 1.8%). The first and third most enriched sequences (2.3 and 1.3%) do not appear to belong to any cluster.

The three most enriched sequences from the two selections were ultimately chosen for further characterization. These were annotated as D1, D2, and D3 for the Dynabeads and C1, C2, and C3 for the SiMAG selections. Additionally, two sequences identified in the two datasets from rounds 23 with preferential abundance in the target pools compared to the counter target pools (sequences 21 and 22 in Table S2 and Figures S3 and S4) were also selected and were annotated as D4, D5, C4, and C5. The sequences of all aptamer candidates are shown in Table S3.

### Screening of the Aptamer Candidates

The 10 selected aptamer candidates were initially evaluated under conditions mimicking the selection process to choose the most promising ones for further analysis. Each aptamer was immobilized on SA-MB via hybridization to a biotinylated docking probe. Aptamer displacing to the solution after incubation with TTX was detected after PCR amplification and agarose gel electrophoresis, as detailed in the Supporting Information. While displacement was observed for all of the aptamer candidates, significant displacement in the presence of TTX was observed for aptamer candidates D3, D4, D5, C2, and C3, which were finally chosen for further evaluation (Figure S5). Moreover, the ssDNA-folding was observed in the predicted structures of the five selected TTX aptamers shown in Figure S7, using the M-fold program (http://www.unafold.org/mfold/applications/dna-folding-form.php.).

### Binding Properties of the Aptamer Candidates

Characterization of the binding properties of aptamers for small-molecular-weight targets like TTX using classical methods is usually hindered by the size of the molecules. A variety of approaches have been reported for affinity studies,^54,75^ including microscale thermophoresis^76,77^ and isothermal titration calorimetry,^51^ but these require specialized equipment. Our group has previously reported the use of magnetic beads for the immobilization of small-molecule targets and the detection of aptamer binding by PCR and colorimetry.^71,72,77^ We have developed microtiter plate-based assays using long-chain crosslinkers to spatially separate the target from the plate surface and facilitate aptamer binding^71,72^ and also used gold nanoparticle aggregation assays.^78^ These methods are easy to perform and require material and equipment found in almost any laboratory.

For the TTX aptamers, three of these methods were exploited. The calculated *K*_D_ values are shown in Table 2, and the respective binding curves in Figure S6. For APAA, TTX was immobilized on magnetic beads, whereas bound unmodified aptamer was detected after PCR amplification and gel electrophoresis. All aptamers demonstrated similar binding affinities with affinity dissociation constants in the range of 73–114 nM. Aptamers C2 and C3 selected using the SiMAG SA-MB showed slightly better *K*_D_ values compared to the ones selected with the Dynabeads SA-MB (D3, D4, and D5). Biotinylated aptamers were used for bead-ELAA in combination with TTX immobilized on magnetic beads. Colorimetric detection of bound aptamers was achieved using SA-pHRP and the TMB substrate. As with APAA, all *K*_D_ values determined with bead-ELAA were calculated in the low nanomolar range (7–89 nM). Given the low nanomolar affinity constants obtained for the five aptamers, all of these were then tested to identify the best aptamer for use in a sandwich assay with the antibody.

**Table 2:** Affinity Dissociation Constants of the Aptamer Candidates Determined by APAA and Bead-ELAA

	APAA		bead-ELAA	
aptamer	*K*_D_ (nM)	*R*^2^	*K*_D_ (nM)	*R*^2^
**D3**	103 ± 24	0.9780	7 ± 1	0.9915
**D4**	96 ± 16	0.9827	29 ± 13	0.9811
**D5**	114 ± 46	0.9435	89 ± 58	0.9570
**C2**	77 ± 6	0.9729	25 ± 6	0.9859
**C3**	73 ± 12	0.9659	29 ± 7	0.9829

### TTX Detection with a Hybrid Antibody–Aptamer Sandwich Assay

**Figure 2:**
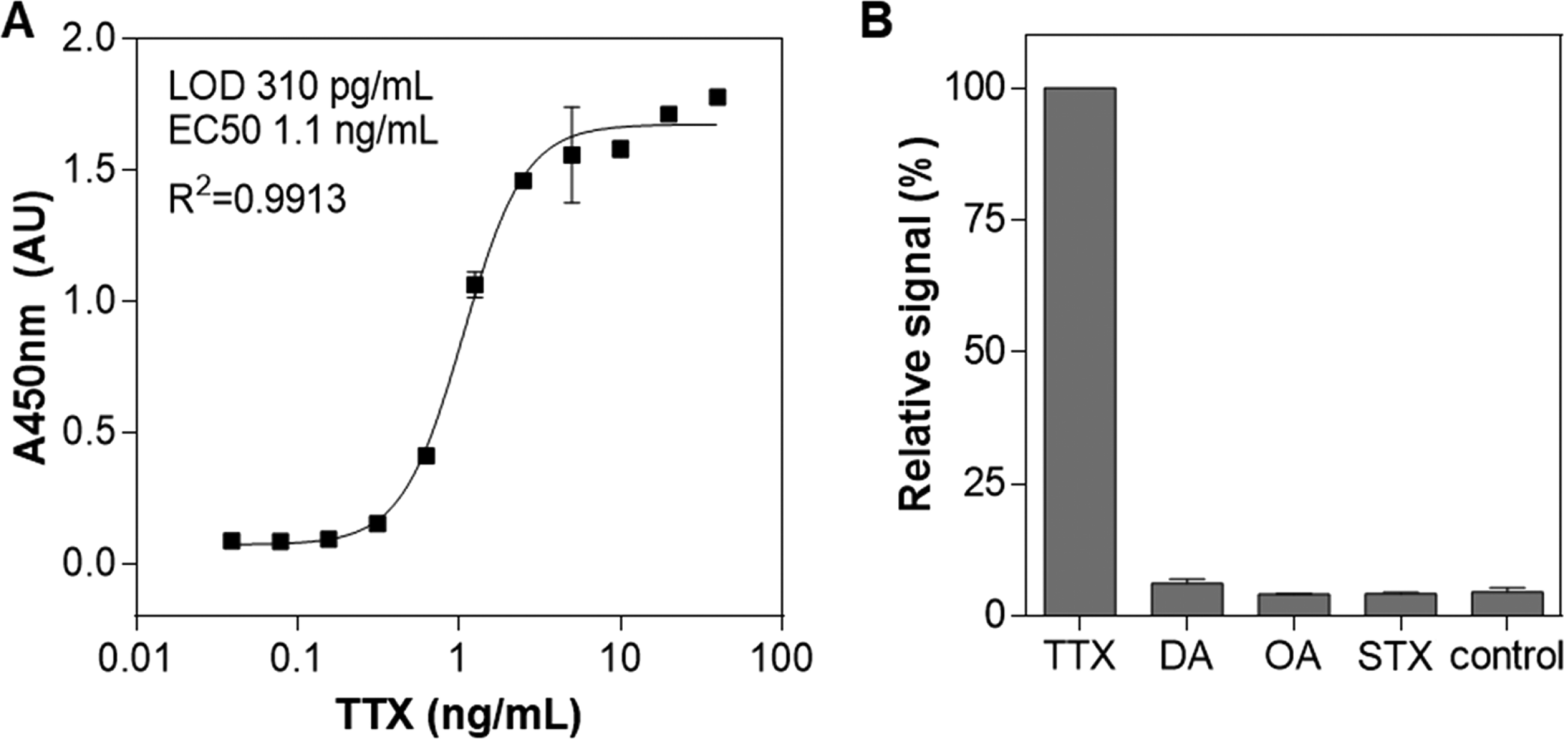
Hybrid antibody–aptamer assay for the detection of TTX. (A) TTX calibration curve with the monoclonal IgG antibody-D3 aptamer pair. (B) Specificity of the assay.

Once the binding properties of the five aptamer candidates were verified, the final objective was to design an aptamer assay for the detection of TTX in relevant samples. Detection of small molecules is usually accomplished with competitive-type assays since the size of the targets usually does not permit the simultaneous binding of more than one biorecognition element. We have previously demonstrated competitive assays using the small molecule target immobilized on magnetic beads^71^ or microplate wells,^72^ and here, we pursued a robust hybrid antibody–aptamer sandwich microtiter plate assay. It was hypothesized that the unique cagelike structure of TTX could potentially allow the formation of an antibody–TTX–aptamer complex, enabling the detection of TTX with a sandwich assay. Even though hybrid antibody–aptamer assays have been reported before for high-molecular-weight targets like proteins and cells,^65^ examples for small-molecule targets are rare. Nevertheless, these assays are very attractive because they combine the advantages of both types of biorecognition elements while at the same time providing the sensitivity/specificity of sandwich assay formats. Using a monoclonal anti-TTX IgG antibody to coat the wells of a microtiter immunoplate, the five TTX aptamers were initially screened to choose the most suitable one for sandwich assay development. Indeed, all aptamers were able to form a sandwich with the antibody and allow the detection of TTX (Figure S8). Aptamer D3, however, was by far the most successful one, leading to more than twofold higher signal compared to the signals obtained with the other aptamers, and it was chosen for final assay development. The sensitivity of the hybrid assay employing the monoclonal TTX antibody for capture and the D3 aptamer for detection was then evaluated at concentrations of TTX ranging from 39 pg/mL to 40 ng/mL, equivalent to 122 pM to 125 nM. The assay was very sensitive with a LOD of 310 pg/mL (970 pM) and EC50 of 1.1 ng/mL or 3.4 nM (Figure 2A). Using TTX samples analyzed on different days, the average interassay coefficients of variation (CV) of less than 5% were calculated, demonstrating the high precision of the assay (Table S4). Finally, the high specificity of the assay was exhibited by the absence of interference from other marine toxins such as domoic acid (DA), okadaic acid (OA), or saxitoxin (STX), the latter sometimes simultaneously present in pufferfish^79^ or shellfish^34^ (Figure 2B). Various assays and biosensors have been reported in the literature for the detection of TTX, and some are summarized in Table S5. To date, the two previously published TTX aptamers have been exploited for the development of fluorescence,^70,78,80^ fluorescence combined with amplification^55^ and electrochemical^81^ assays, and the LODs achieved ranged from 0.265 pg/mL to 319 ng/mL. Competitive immunoassays have also been reported using monoclonal TTX antibodies,^51,52,82^ and their sensitivity was 0.3–2.5 ng/mL. The performance of the assay developed in this work is therefore superior or at least comparable to many of the previously published assays employing aptamers or antibodies. Very importantly, the majority of previously reported assays are quite complicated to perform as opposed to the simple sandwich assay demonstrated in this work. Commercial TTX kits are available, and they are based on competitive immunoassays. Examples include the microplate kits from CD Creative Diagnostics and United Biotechnology with LODs of 1–10 ng/mL, as well as the rapid lateral flow tests from CD Creative Diagnostics and UNIBIOTEST with a sensitivity of 0.1–2 μg/mL. It is thus evident that the hybrid antibody–aptamer format of the assay described herein has great potential for use in lateral flow tests, facilitating the facile and rapid on-site detection of TTX in field samples, especially when combined with a simple method for sample preparation. It is also one of the rare examples of such hybrid assays for the detection of a small-molecular-weight analyte since there are reports for only three other targets, trinitrotoluene,^66^ tetracycline,^67^ and aflatoxin B1.^68^

### Application of the Assay to Pufferfish Analysis

The hybrid antibody–aptamer sandwich assay was finally employed for the analysis of field samples. Extracts from different tissues (gonads, liver, skin, and muscle) of *L. lagocephalus* pufferfish were prepared as controls since our previous report showed the absence of TTX in these tissues.^73^ The extracts were diluted, spiked with TTX, and analyzed with the assay as explained in the Experimental Section. As shown in Table 3, excellent recoveries were achieved in the range of 93.5–109.1%, thus demonstrating the absence of matrix effects and the compatibility of the assay with such samples. Extracts from tissues of *L. sceleratus* pufferfish previously shown to contain high levels of TTX^73^ were then analyzed. Since TTX may coexist with several other naturally occurring TTX analogues, the hybrid sandwich assay is expected to provide a global TTX response depending on the specificity of both the antibody and aptamer. The TTX content was determined using calibration curves constructed both in PBS (after applying the corresponding recovery factor) and in the respective tissue extract from the TTX-free pufferfish. As expected, TTX contents with both strategies were very similar. High TTX levels were observed, especially in the gonads and liver tissues where TTX usually bioaccumulates (Table 3). The TTX content in these tissues was 2.5–5-fold higher than the permissible levels in Japan (2 mg of TTXs/kg). For comparison, the samples were analyzed in parallel with a competitive magnetic bead-based ELISA (detailed in the SI), which was previously developed and exploited a different monoclonal antibody.^23,83^ Some differences were observed, which may derive from the specificity of the assays toward the different TTX analogues. It is necessary to take into account that the cross-reactivity factors for the different TTX analogues may vary according to the biorecognition molecule (which in the case of the hybrid sandwich assay are both the antibody and the aptamer) and also the format of the assay. Nevertheless, comparable results were obtained with both methods. A very good correlation was also observed with a previous analysis carried out with LC-MS/MS;^73^ the TTX contents trend in the different tissues are the same: gonads > liver > skin > mussel (Table S6). The establishment of the cross-reactivity factors for the different TTX analogues present in these tissues would facilitate the comparison with LC-MS/MS results. However, pure TTX analogues are not commercially available, and their production is not a facile task. The isolation of the TTX analogues and the elucidation of the toxicity equivalence factors, alongside the cross-reactivity factors, which ideally should be similar, is part of ongoing work.

**Table 3:** Detection of TTX in Pufferfish Extracts

		TTXs content (*L. sceleratus*)^b^(mg TTX equiv/kg)		
		hybrid antibody–aptamer sandwich assay		
tissue	% TTX recovery (*L. lagocephalus*)^a^	PBS	extract	competitive magnetic bead ELISA
gonads	109.1	9.46	9.94	5.24
liver	93.5	5.99	5.01	2.84
skin	107.7	0.98	1.28	0.19
muscle	96.3	0.86	0.82	0.42

a Recovery (%) of TTX spiked in diluted extracts from a TTX-free fish (*L. lagocephalus*).

b TTX content (mg TTX equiv/kg of tissue) in extracts from a TTX-containing fish (*L. sceleratus*) was determined using calibration curves constructed in PBS buffer and in the respective extract from the TTX-free fish.

## Conclusions

TTX has emerged as a major food hazard because of its high neurotoxicity and its presence in seafood found not only in Asian but also European waters. Traditionally, bioassays have been used to detect TTX; however, instrumental analysis using liquid chromatography in combination with mass spectrometry is currently employed for monitoring field samples. Microplate immunoassays and antibody-based biosensors can also provide the required sensitivity and specificity, provided that highly specific antibodies are used. Aptamers are cost-effective alternatives to monoclonal antibodies, and since their discovery in the early 1990s, they have been used for the detection of not only large targets such as cells and proteins but also small-molecular-weight targets like toxins. To date, only two TTX-specific aptamers have been reported and have been exploited for the development of fluorescence and electrochemical assays, which are quite elaborate and are not compatible with the rapid and facile on-site analysis and have not been employed for the analysis of field samples. In this work, capture-SELEX technology in combination with high-throughput NGS analysis was exploited for the discovery of TTX aptamers. Assays using magnetic beads were developed for the verification of the binding properties of the selected aptamer candidates, which exhibited *K*_D_ values in the low nanomolar range. The specific properties of the streptavidin magnetic beads used to immobilize the library and perform the two parallel selections appeared to affect the speed of evolution and the enrichment achieved, although the binding properties of the selected aptamers were not significantly affected. Finally, a simple hybrid antibody–aptamer sandwich assay was demonstrated with high sensitivity, precision, and specificity. Its sensitivity was superior or at least comparable to commercial kits based on competitive immunoassays and other existing aptamer and antibody-based assays and biosensors. The excellent performance of the assay was further demonstrated by the reliable determination of TTX levels in pufferfish with an excellent degree of correlation with measurements obtained with a competitive magnetic bead-based immunoassay and liquid chromatography-mass spectrometry. This is the first demonstration of an assay employing an aptamer for the detection of TTX in pufferfish, and, in general, is one of the very few examples reported in the literature of such hybrid antibody–aptamer sandwich assay for small-molecular-weight analytes. The sandwich format of the assay is particularly attractive, and ongoing work is focused on its transfer to a lateral flow assay to allow the rapid and facile analysis of samples at the point of need. The evaluation of cross-reactivity factors for different TTX analogues with this hybrid antibody–aptamer assay, as well as its applicability to the analysis of shellfish, is also in progress.

## Supporting Information Available

Conditions used for the selections; NGS data bioinformatics analysis; characterization of aptamer candidates; and TTX detection (PDF)
