# Supplementary material for: Hybrid Antibody–Aptamer Assay for Detection of Tetrodotoxin in Pufferfish
Source: Anal Chem. 2021 Oct 26;93(44):14810–9. doi: 10.1021/acs.analchem.1c03671 (PMC8581965; doi:10.1021/acs.analchem.1c03671)
Supplement: Supplementary file 1 — ac1c03671_si_001.pdf [file ac1c03671_si_001.pdf]

SUPPLEMENTARY INFORMATION

Hybrid antibody-aptamer assay for detection of tetrodotoxin in puffer fish

Xhensila Shkembi[1], Vasso Skouridou[1], Marketa Svobodova[1], Sandra Leonardo[2], Abdulaziz S. Bashammakh[3], Abdulrahman O. Alyoubi[3], Mònica Campàs[2*], Ciara K. O´Sullivan[1,4*]

[1] *Interfibio, Nanobiotechnology and Bioanalysis Group, Departament d'Enginyeria Química, Universitat Rovira i Virgili, Avinguda Paisos Catalans 26, 43007 Tarragona, Spain*

[2] *IRTA, Ctra Poble Nou km 5.5, 43540 Sant Carles de la Ràpita, Spain*

[3] *Department of Chemistry, Faculty of Science, King Abdulaziz University, P.O. Box 80203, 21589 Jeddah, Kingdom of Saudi Arabia*

[4] *Institució Catalana de Recerca I Estudis Avancats (ICREA), Passeig Lluís Companys 23, 08010 Barcelona, Spain*

* Corresponding author: ciara.osullivan@urv.cat, tel.: +34977558740; monica.campas@irta.cat

Table of contents

1. Selection process S2

Table S1. Conditions used for the selections. S3

2. NGS analysis S4

Table S2. Distribution (%) of highly abundant sequences in the different pools from the two selections. Sequences were ranked according to their abundance in the TTX pool from round 23. ....S4

Figure S1. ....S5

Figure S2. Abundance of highly abundant sequences in the last selection round of the target and counter selection molecules pools using SiMAG SA-MB for library immobilization. S5

Figure S3. Multiple sequence alignment of the 100 most abundant sequences in the TTX target pool from round 23 of the selection performed with the Dynabeads SA-MB. Identical bases are shaded, and the three most enriched sequences selected for characterization are in boxes. ...S7

Figure S4. Multiple sequence alignment of the 100 most abundant sequences in the TTX target pool from round 23 of the selection performed with the SiMAG SA-MB. Identical bases are shaded and the three most enriched sequences selected for characterization are in boxes. S9

Table S3. Sequences of the selected aptamer candidates. D sequences were identified from the selection with Dynabeads and C sequences with the SiMAG SA-MB. S10

3. Characterization of aptamer candidates S10

3.1 Immobilization of TTX on magnetic beads for affinity assays S10

3.2 Initial screening of the aptamer candidates S11

Figure S5. Screening of the aptamer candidates with a displacement assay. Aptamer candidates immobilized on docking probe-streptavidin magnetic beads complexes were incubated with 100

µM TTX (+) or only binding buffer (-). Aptamer displacing to the solution was detected by PCR amplification and agarose gel electrophoresis. ntc: PCR no template control.........................S11

Figure S6. Binding curves of the aptamers determined by (A) APAA and (B) bead-ELAA......S12

Figure S7. Predicted structures of the five selected TTX aptamers..S12

4. TTX detection S13

Figure S8. Screening of antibody-aptamer pairs for sandwich assay development. S13

Table S4. Hybrid antibody-aptamer assay precision. Inter-assay coefficients of variation (% CV) were calculated from duplicate samples using solutions of different TTX concentration measured on four different days (n=4)..S13

Table S5. Assays and biosensors reported in the literature for TTX detection. S15

5. References..S15

1. Selection process

For library immobilization on magnetic beads, the biotinylated docking probe was first captured on the streptavidin magnetic beads (SA-MB) according to the manufacturer's instructions, using a 1.5-fold molar excess of the docking probe over the theoretical binding capacity of the beads. The library and the subsequent pools, prepared in binding buffer (BB, PBS with 1.5 mM $MgCl_2$), were annealed (2 min at 95ºC and slow cooling to 4ºC) and then incubated overnight at 22ºC under rotation for immobilization on the SA-MB/docking probe complexes. For the first round, 100 µL of SA-MB were used for the immobilization of 0.2 nmol of the library whereas for subsequent rounds, 20 – 40 µL of SA-MB were required according to the amount of ssDNA pool prepared at the end of each round. Unbound oligonucleotides were removed by extensive washing of the beads (5 x 500 µL of binding buffer) and the SA-MB/docking probe/library complexes were finally resuspended with binding buffer to 10 mg/mL. Selection was initiated with a temperature pre-elution step for 15 min at 28ºC under gentle agitation. After removal of the supernatant, the beads were resuspended in the same volume of binding buffer and a background elution step was performed. Finally, the beads were resuspended in the same volume of binding buffer containing tetrodotoxin for the target elution step, which was performed under the same conditions as the background elution to select sequences eluting in the presence of the target molecule. During the last six rounds, tetrodotoxin precursors L-arginine and 1,6-anhydro-beta-d-mannopyranose were included during the background elution step as counter-selection molecules. A total of 23 rounds were performed and the specific conditions used for each round are shown in Table S1 (Supplementary Information). The supernatants from the three elution steps were collected via magnetic separation and were used for pilot PCR experiments to monitor the progress of the selections. To this end, 2 µL of each fraction (pre-elution, background elution and target elution) were added to 10 µL of PCR master mix containing library-specific forward and phosphorylated reverse primers. Pilot PCR was performed using an initial denaturation step of 2 min at 95°C followed by 6 – 16 cycles of (15 sec at 95°C, 15 sec at 55°C, 30 sec at 72°C). For the preparation of ssDNA for succeeding rounds, PCR reactions were performed using the optimal number of

amplification cycles found by pilot PCR, followed by asymmetric PCR and lambda exonuclease digestion. For asymmetric PCR, the PCR master mix contained only the forward primer and 20 µL of PCR reaction per 100 µL of master mix and amplification was performed for 12 cycles. Remaining dsDNA in the asymmetric PCR reaction was digested by lambda exonuclease for 1.5 h at 37°C, followed by enzyme deactivation for 10 min at 80ºC and column purification of the generated ssDNA for use in subsequent rounds of selection.

Table S1. Conditions used for the selections.

Selection round	Duration* (min)	Tetrodotoxin (µM)	Counter-selection
R1 – R4	120	500	no
R5	120	100	no
R6	120	100	no
R7 – R13	60	100	no
R14 – R16	30	100	no
R17 – R23	30	50	yes **

** background, counter and target elution steps*

*** L-arginine and 1,6-anhydro-β-D-mannopyranose*

2. NGS analysis

Table S2. Distribution (%) of highly abundant sequences in the different pools from the two selections. Sequences were ranked according to their abundance in the TTX pool from round 23.

Sequence		Dynabeads SA-MB			SiMAG SA-MB		
		R23	R23-counter	R16	R23	R23-counter	R16
Most abundant	1	2.125	2.373	0.095	2.321	1.438	0.041
	2	1.138	1.351	0.063	1.825	1.478	0.061
	3	0.884	0.873	0.197	1.308	1.062	0.040
	4	0.477	0.492	0.016	0.985	0.841	0.014
	5	0.436	0.435	0.137	0.768	0.371	0.020
	6	0.359	0.381	0.051	0.666	0.448	0.009
	7	0.314	0.321	0.035	0.535	0.474	0.056
	8	0.311	0.332	0.034	0.459	0.363	0.017
	9	0.285	0.366	0.007	0.359	0.259	0.005
	10	0.263	0.298	0.012	0.354	0.284	0.034
	11	0.250	0.289	0.102	0.277	0.211	0.012
	12	0.244	0.384	0.007	0.270	0.231	0.003
	13	0.228	0.335	0.007	0.268	0.175	0.000
	14	0.224	0.226	0.009	0.235	0.109	0.011
	15	0.192	0.163	0.003	0.228	0.135	0.002
	16	0.192	0.160	0.028	0.191	0.165	0.000
	17	0.179	0.232	0.003	0.184	0.122	0.026
	18	0.179	0.169	0.060	0.184	0.150	0.023
	19	0.176	0.166	0.009	0.163	0.126	0.006
	20	0.176	0.143	0.021	0.158	0.113	0.008
More abundant in target pool	21	0.087	0.031	0.007	0.028	0.007	0.000
	22	0.026	0.000	0.000	0.026	0.005	0.000

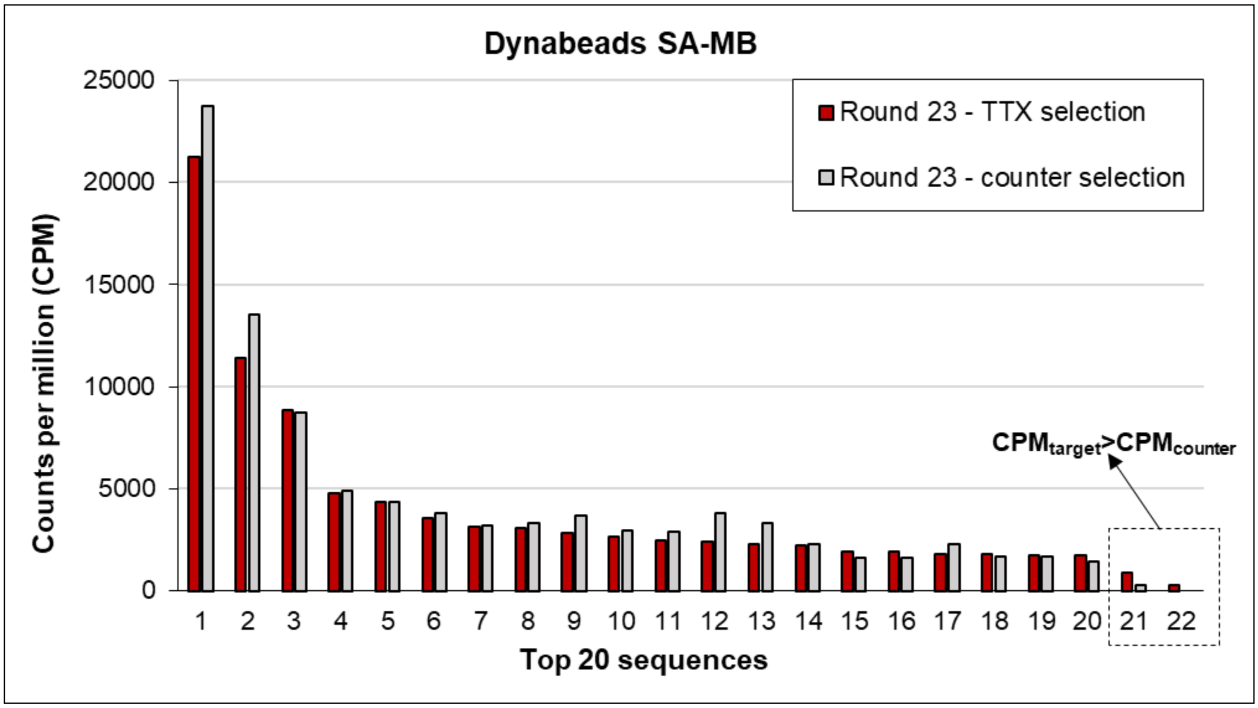

Figure S1. Abundance of highly abundant sequences in the last selection round of the target and counter selection molecules pools using Dynabeads SA-MB for library immobilization

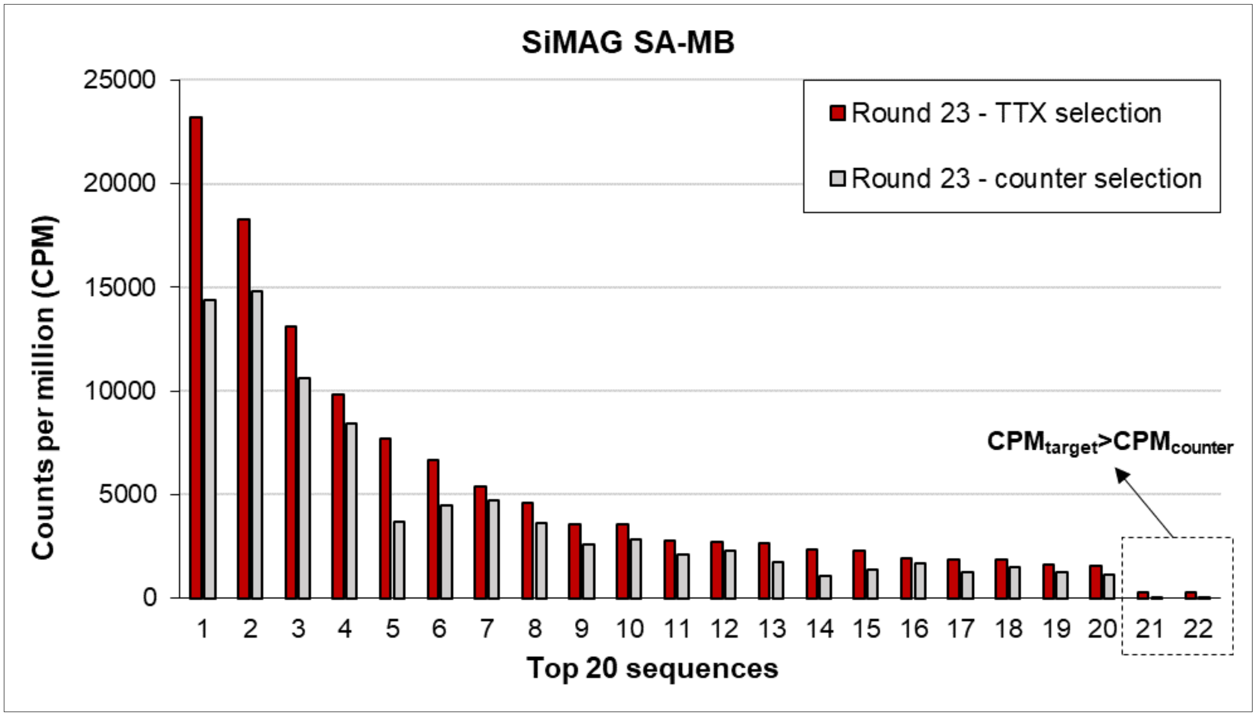

Figure S2. Abundance of highly abundant sequences in the last selection round of the target and counter selection molecules pools using SiMAG SA-MB for library immobilization.

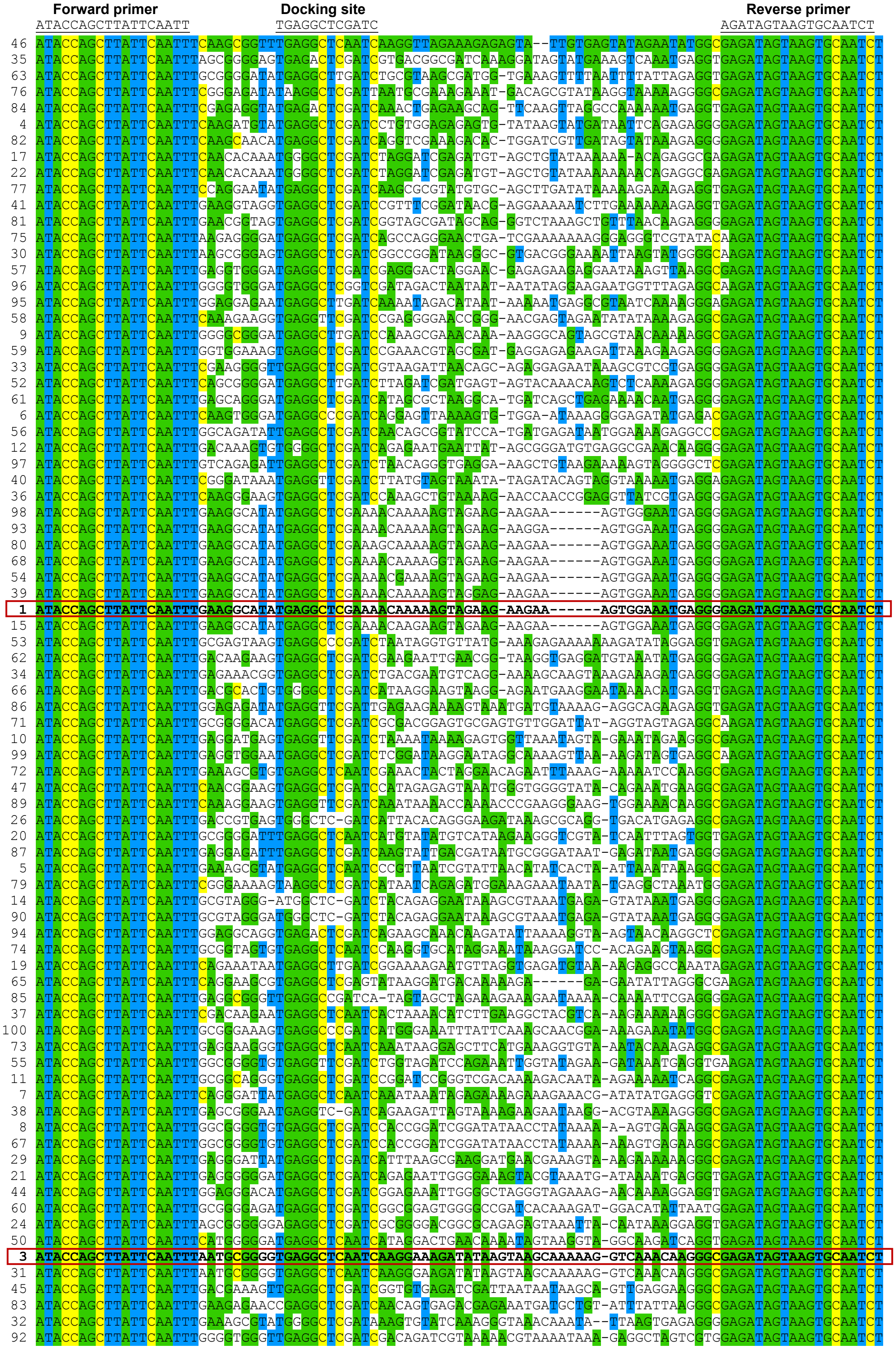
Forward primer
ATACCAGCTTATTCAATT
Docking site
TGAGGCTCGATC
Reverse primer
AGATAGTAAGTGCAATCT

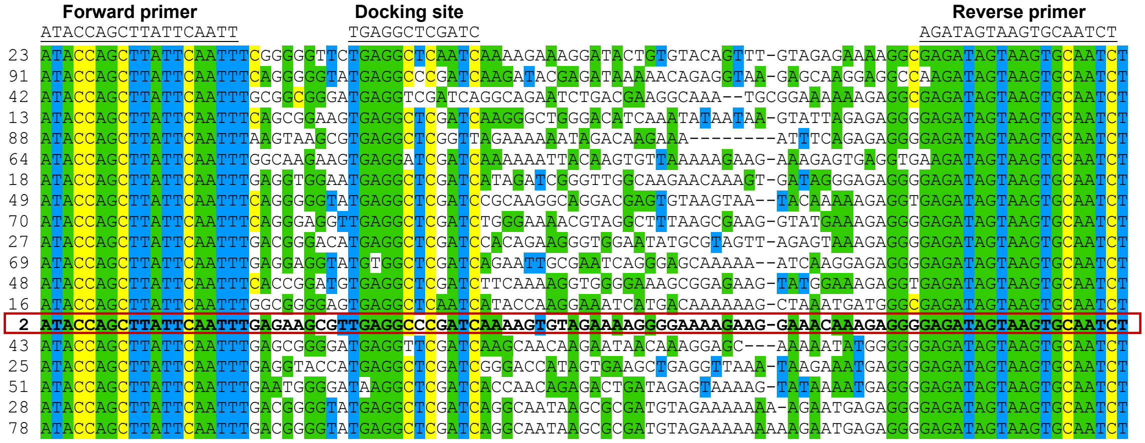

Figure S3. Multiple sequence alignment of the 100 most abundant sequences in the TTX target pool from round 23 of the selection performed with the Dynabeads SA-MB. Identical bases are shaded, and the three most enriched sequences selected for characterization are in boxes.

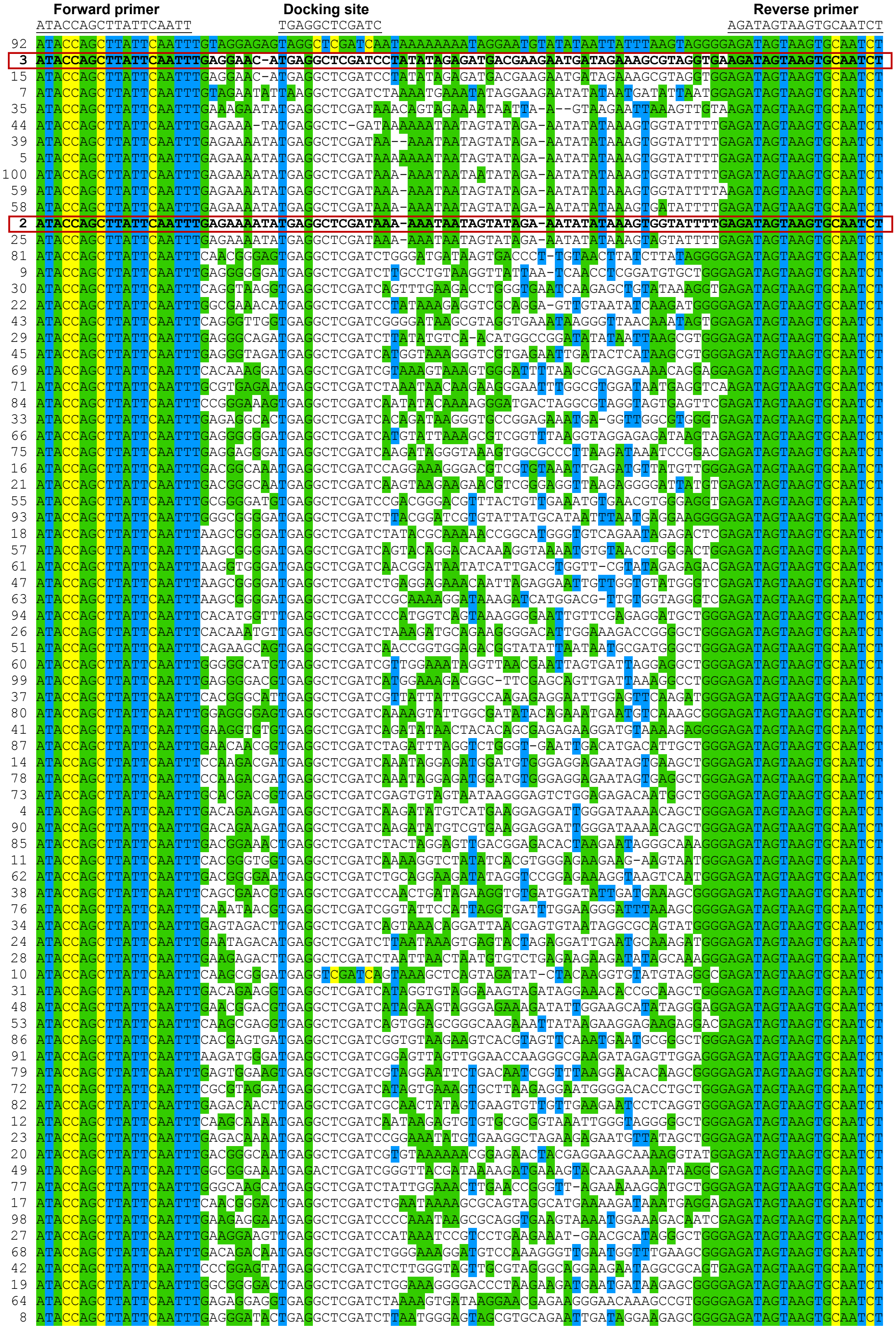

Forward primer
ATACCAGCTTATTCAATT
Docking site
TGAGGCTCGATC
Reverse primer
AGATAGTAAGTGCAATCT

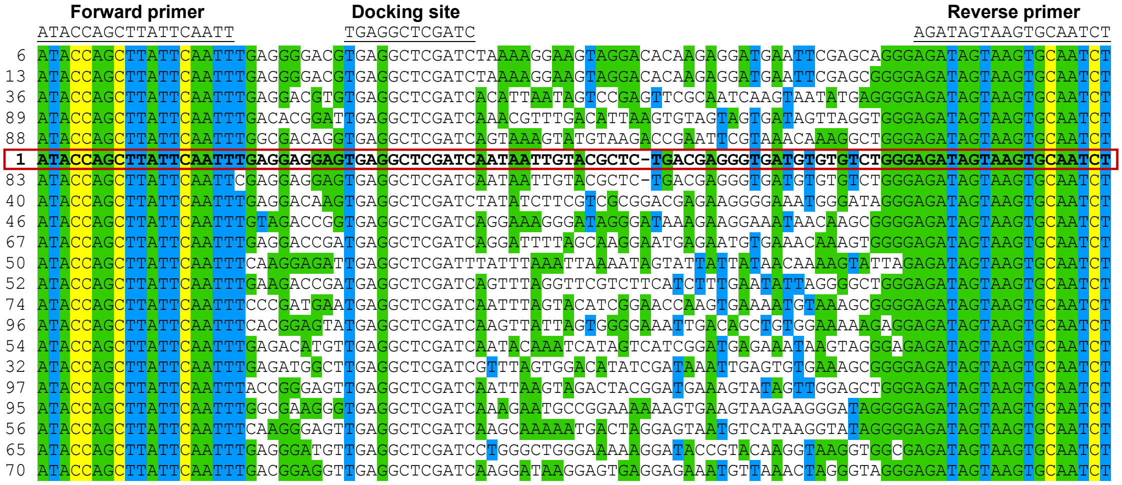

Figure S4. Multiple sequence alignment of the 100 most abundant sequences in the TTX target pool from round 23 of the selection performed with the SiMAG SA-MB. Identical bases are shaded and the three most enriched sequences selected for characterization are in boxes.

Table S3. Sequences of the selected aptamer candidates. D sequences were identified from the selection with Dynabeads and C sequences with the SiMAG SA-MB.

ID	Sequence (5'-3')	Length (nt)	GC (%)
D1	ATACCAGCTTATTCAATTTGAAGGCATATGAGGCTCGAAAACAAAAAGTAGAAGAAGAAAGTGGAAATGAGGGGAGATAGTAAGTGCAATCT	92	37.0
D2	ATACCAGCTTATTCAATTTGAGAAGCGTTGAGGCCCGATCAAAAGTGTAGAAAAGGGGAAAAGAAGGAAACAAAGAGGGGAGATAGTAAGTGCAATCT	98	40.8
D3	ATACCAGCTTATTCAATTTAATGCGGGGTGAGGCTCAATCAAGGAAAGATATAAGTAAGCAAAAAGGTCAAACAAGGGCGAGATAGTAAGTGCAATCT	98	38.8
D4	ATACCAGCTTATTCAATTTGAGGAGGTATGTGGCTCGATCAGAATTGCGAATCAGGGAGCAAAAAATCAAGGAGAGGGGAGATAGTAAGTGCAATCT	97	42.3
D5	ATACCAGCTTATTCAATTTGAGCGTGGGTGAGGCTTGATCCGAGGGTAGTTAGCGTAGCGAAGGAAGAAAAAAAGAGGGGAGATAGTAAGTGCAATCT	98	44.9
C1	ATACCAGCTTATTCAATTTGAGGAGGAGTGAGGCTCGATCAATAATTGTACGCTCTGACGAGGGTGATGTGTGTCTGGGAGATAGTAAGTGCAATCT	97	44.3
C2	ATACCAGCTTATTCAATTTGAGAAAATATGAGGCTCGATAAAAAATAATAGTATAGAAATATATAAAGTGGTATTTTGAGATAGTAAGTGCAATCT	96	26.0
C3	ATACCAGCTTATTCAATTTGAGGAACATGAGGCTCGATCCTATATAGAGATGACGAAGAATGATAGAAAGCGTAGGTGAAGATAGTAAGTGCAATCT	97	38.1
C4	ATACCAGCTTATTCAATTTCACGGGGGGTGAGGCTCGATCTGTAATTAAGAGTGCAAGGGGAAGTGAGATGAAAGTTGGGAGATAGTAAGTGCAATCT	98	43.9
C5	ATACCAGCTTATTCAATTTGAGGCGAGGTGAGGCTCGATCAATAGAAAAACCGAGGCGAAAATGAGAAAAAGGGACTGGGAGATAGTAAGTGCAATCT	98	42.9

3. Characterization of aptamer candidates

3.1 Immobilization of TTX on magnetic beads for affinity assays

TTX was immobilized on magnetic as follows: maleimide-activated magnetic beads (10 µL of 250 mg/mL suspension) were washed with washing buffer (PBST: 0.1 M PBS, 0.05 % v/v Tween-20, pH 7.2), resuspended in 500 µL of 1 mM MUAM in binding buffer (0.1 M PBS, pH 7.2, 10 % v/v ethanol) and incubated for 3 h at room temperature under tilt rotation. The beads were washed again with PBST, followed by resuspension with 500 µL of TTX (25 µg/mL in 0.1 M PBS, 10 % v/v formaldehyde). After overnight incubation at ambient temperature (22-25ºC) under tilt rotation, the beads were washed again and blocked with 500 µL of sulfo-NHS-acetate (1 mM in 0.1 M PBS)

for 1 h. After a final washing step, the TTX-beads were resuspended in 100 µL of PBS (final suspension of 25 mg/mL). Immobilization of TTX on the beads was verified with a bead-ELISA using a monoclonal anti-TTX antibody as described previously[1].

3.2 Initial screening of the aptamer candidates

A displacement assay was designed to screen the aptamer candidates under conditions similar to the ones used during the selection process. Specifically, SiMAG SA-MB/docking probe complexes were prepared as described in the "Capture-SELEX process" section of the manuscript and used to immobilize the individual aptamer candidates (100 nM). The aptamer-magnetic beads were then incubated with TTX (1 µM) or binding buffer alone for 30 min at room temperature under tilt rotation. The supernatant was recovered by magnetic separation and was used for PCR amplification to detect eluted sequences. The PCR reactions were analyzed by agarose gel electrophoresis as shown in Figure S5. The candidates preferentially eluting in the presence of TTX compared to buffer alone were selected for characterization of their binding properties.

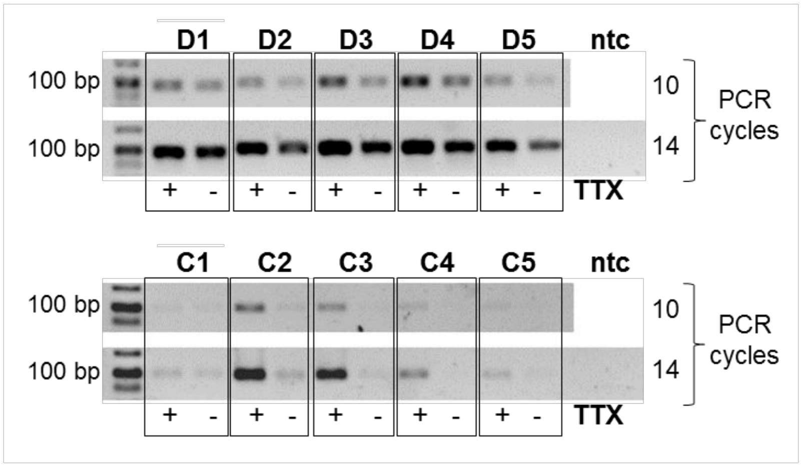

Figure S5. Screening of the aptamer candidates with a displacement assay. Aptamer candidates immobilized on docking probe-streptavidin magnetic beads complexes were incubated with 100 µM TTX (+) or only binding buffer (-). Aptamer displacing to the solution was detected by PCR amplification and agarose gel electrophoresis. ntc: PCR no template control.

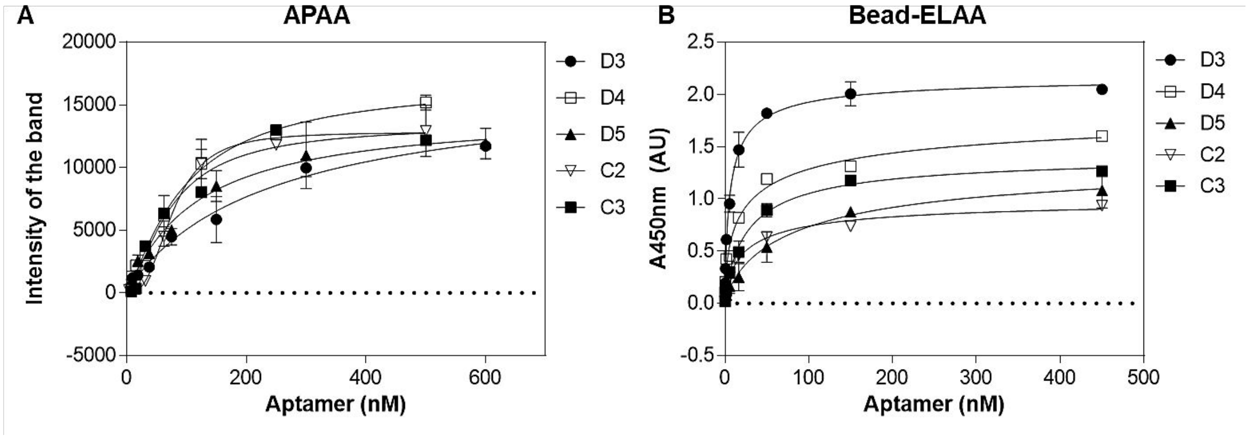

Figure S6. Binding curves of the aptamers determined by (A) APAA and (B) bead-ELAA

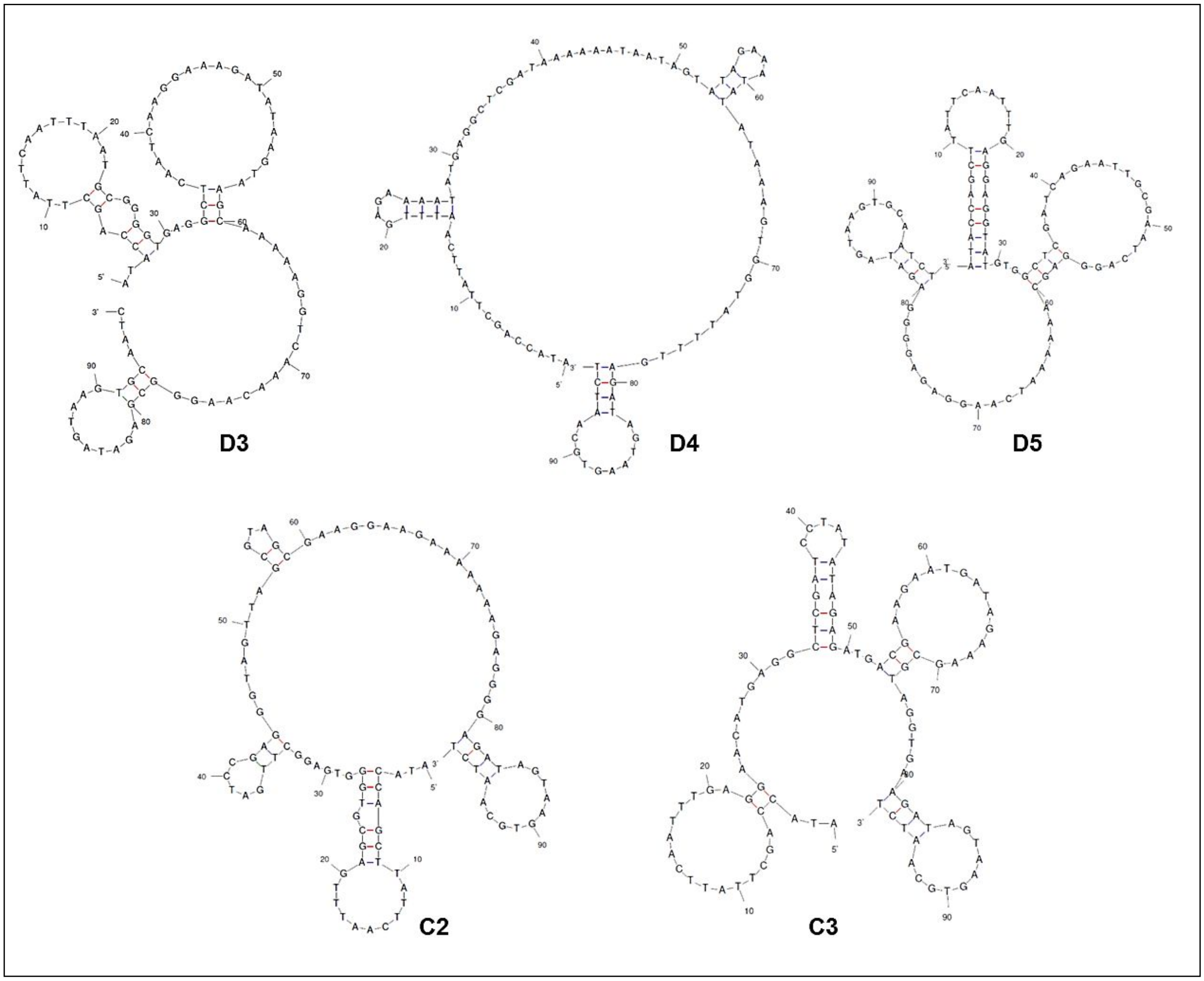

Figure S7. Predicted structures of the five selected TTX aptamers.

4. TTX detection

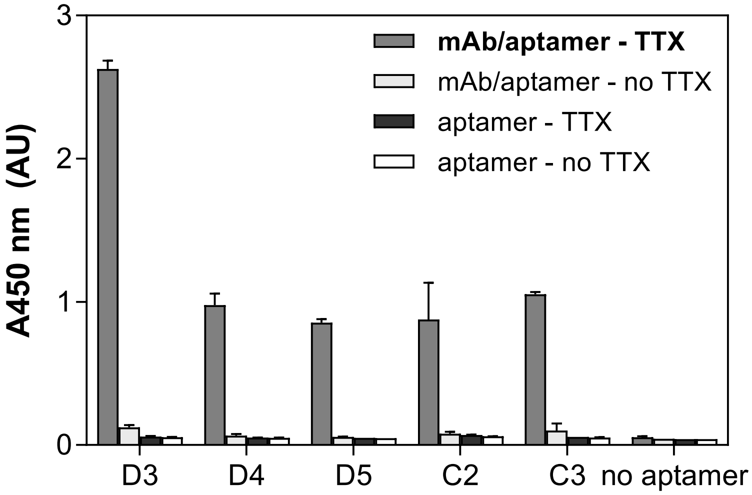

Figure S8. Screening of antibody-aptamer pairs for sandwich assay development.

Table S4. Hybrid antibody-aptamer assay precision. Inter-assay coefficients of variation (% CV) were calculated from duplicate samples using solutions of different TTX concentration measured on four different days (n=4).

Sample	Concentration (ng/mL)	MV ± SD	% CV	Average % CV
1	1.25	2.420 ± 0.077	3.2	2.8
		2.416 ± 0.003	0.1	
		2.357 ± 0.069	2.9	
		2.334 ± 0.112	4.8	
2	0.625	1.371 ± 0.064	4.9	3.3
		1.702 ± 0.007	0.4	
		1.219 ± 0.057	4.7	
		1.615 ± 0.055	3.4	
3	0.3125	0.443 ± 0.009	2.0	4.3
		0.782 ± 0.014	1.8	
		0.361 ± 0.002	0.5	
		0.436 ± 0.056	12.8	
4	0.039	0.157 ± 0.001	0.6	2.9
		0.160 ± 0.011	6.7	
		0.095 ± 0.004	4.1	
		0.145 ± 0.000	0.2	

4.1 Magnetic bead-based colorimetric immunoassay for TTX detection

The MB-based colorimetric immunoassay protocol was similar to that described[2] and optimized[3] in our previous works. Briefly: (1) 10 µL of maleimide-activated MBs were rinsed three times with washing buffer (0.1 M PBS, 0.05 % v/v Tween−20, pH 7.2) under vigorous mixing; (2) 1 mL of 1 mM cysteamine in binding buffer (0.1 M PBS, 10 mM EDTA, pH 7.2) was added and incubated for 2 h at room temperature; (3) after washing, 1 mL of TTX solution (25 µg/mL) in binding buffer containing 10 % v/v formaldehyde was added and incubated overnight at 4°C; (4) the washed TTX-coated MBs were resuspended in 1 mL of binding buffer. When amounts of MB varied, volumes were adjusted proportionally. Once the MB-TTX conjugate had been prepared, (5) 200 µL of the conjugate was taken, the supernatant was removed and 100 µL of the TTX standard solution or fish extract and 100 µL of anti-TTX mAb at 1/2000 dilution in 1 % w/v BSA-binding buffer were added and incubated for 30 min at room temperature; (6) after washing, 200 µL of 1/1000 IgG-HRP dilution in 1 % w/v BSA-binding buffer was incubated for 30 min at room temperature; (7) the washed immunocomplex was resuspended in 200 µL of binding buffer; (8) 50 µL of immunocomplex was transferred to a new tube and after supernatant removal, 125 µL of TMB liquid substrate was added and incubated for 10 min; (9) the tube was placed on the magnetic separation stand and 100 µL of TMB liquid substrate was collected for colorimetric measurement at 620 nm in a microtiter plate. All incubation steps were performed under agitation. Measurements were performed in triplicate.

Table S5. Assays and biosensors reported in the literature for TTX detection.

Platform	Sensitivity (LOD)	Reference
Fluorescence assay with aptamer	1 µM (319 ng/mL)	4
Electrochemical impedance spectroscopy biosensor with aptamer immobilized on glassy carbon electrode	200 pg/mL	5
Fluorescence assay with aptamer and berberine	0.074 nM (24 pg/mL)	6
Fluorescence assay with aptamer, berberine and exonuclease I	11 pM (3.5 pg/mL)	7
Fluorescence assay with FAM-labeled aptamer and magnetic reduced graphene oxide	1.21 ng/mL	8
Competitive assay with aptamer immobilized on magnetic beads, strand displacement amplification with catalytic hairpin assembly and fluorescence detection	0.265 pg/mL	9
Microplate competitive immunoassay	2.28 ng/mL	10
Competitive planar wavelength immunosensor	2.5 ng/mL	11
Inhibition immunoassay with surface plasmon resonance sensor	0.3 ng/mL	12
Hybrid antibody-aptamer sandwich assay	310 pg/mL (970 pM)	This work

Table S6. TTX and analogues contents (mg TTX or analogue/kg tissue) in *L. sceleratus* by LC-MS/MS.

	TTX	4-*epi*TTX	11-norTTX-6(*R*)-ol	11-norTTX-6(*S*)-ol	4,9-anhydroTTX	5-deoxyTTX	11-deoxyTTX	5,11-dideoxyTTX/ 6,11-dideoxyTTX	5,6,11-trideoxyTTX
Gonads	21.8	4.3	1.1	16.3	0.5	0.9	1.1	0.4	94.3
Liver	2.3	0.7	0.3	1.3	0.2	-	0.2	0.2	12.4
Skin	1.2	0.3	0.1	1.1	-	-	0.1	-	1.8
Muscle	0.7	0.3	0.2	0.6	0.1	-	0.1	0.1	1.2

5. References

1. Skouridou, V.; Jauset-Rubio, M.; Ballester, P.; Bashammakh, A. S.; El-Shahawi, M. S.; Alyoubi, A. O.; O'Sullivan, C. K. Selection and Characterization of DNA Aptamers Against the Steroid Testosterone. *Microchim. Acta* **2017**, *184*, 1631-1639.
2. Leonardo, S.; Kiparissis, S.; Rambla-Alegre, M.; Almarza, S.; Roque, A.; Andree, K.B.; Christidis, A.; Flores, C.; Caixach, J.; Campbell, K.; Elliott, C.T.; Aligizaki, K.; Diogène, J.; Campàs, M. Detection of tetrodotoxins in juvenile pufferfish Lagocephalus sceleratus (Gmelin, 1789) from the North Aegean Sea

(Greece) by an electrochemical magnetic bead-based immunosensing tool. *Food Chem.* **2019**, *290*, 255–262.

3. Campàs, M.; Reverté, J.; Rambla-Alegre, M.; Campbell, K.; Gerssen, A.; Diogène, J. A fast magnetic bead-based colorimetric immunoassay for the detection of tetrodotoxins in shellfish. *Food Chem. Toxicol.* **2020**, *140*, 111315.
4. Shao, B. Y.; Chen, B.; Chen, W.B.; Yang, F.; Miao, T.Y.; Peng J. Preparation and Application of Tetrodotoxin DNA Aptamer. *J. Food Sci.,* **2014**, *35*, 205-208.
5. Fomo, G.; Waryo, T.; Sunday, C.; Baleg, A. A.; Baker, P.; Iwuoha, E. Aptameric Recognition-Modulated Electroactivity of Poly/4-Styrenesolfonic Acid)-Doped Polyaniline Films for Single-shot Detection of Tetrodotoxin. *Sensors* **2015**, *15*, 22547-22560.
6. Lan, Y.; Qin, G.; Wei, Y.; Dong, C.; Wang, L. Highly Sensitive Analysis of Tetrodotoxin Based on Free-Label Fluorescence Aptamer Sensing System. *Spectrochim. Acta A Mol. Biomol. Spectrosc.* **2019**, *219*, 411-418.
7. Lan, Y.; Qin, G.; Wei, Y.; Wang, L.; Dong, C. Exonuclease I-assisted Fluorescence Aptasensor for Tetrodotoxin. *Ecotoxicol. Environ. Saf.* **2020**, *194*, 110417.
8. Gu, H.; Duan, N.; Xia, Y.; Hun, X.; Wang, H.; Wang, Z. Magnetic Separation-based Multiple SELEX for Effectively Selecting Aptamers Against Saxitoxin, Domoic acid and Tetrodotoxin. *J. Agric. Food Chem.* **2018**, *66*, 9801-9809.
9. Zhang, M.; Wang, Y.; Wu, P.; Wang, W.; Cheng, Y.; Huang, L.; Bai, J.; Peng, Y.; Ning, B.; Gao, Z.; Liu, B. Development of a highly sensitive detection method for TTX based on a magnetic bead-aptamer competition system under triple cycle amplification. *Anal. Chim. Acta* **2020**, *1119*, 18-24.
10. Reverté, L.; de la Iglesia, P.; del Río, V.; Campbell, K.; Elliott, C. T.; Kawatsu, K.; Katikou, P.; Diogène, J.; Campàs, M. Detection of Tetrodotoxins in Puffer Fish by a Self-Assembled Monolayer-Based Immunoassay and Comparison with Surface Plasmon Resonance, LC-MS/MS, and Mouse Bioassay. *Anal. Chem.* **2015**, *87*, 10839-10847.
11. Reverté, L.; Campàs, M.; Yakes, B. J.; Deeds, J. R.; Katikou, P.; Kawatsu, K.; Lochhead, M.; Elliott, C. T.; Campbell, K. Tetrodotoxin Detection in Puffer Fish by a Sensitive Planar Waveguide Immunosensor. *Sens. Actuators B Chem.* **2017**, *253*, 967-976.
12. Taylor, A. D.; Ladd, J.; Etheridge, S.; Deeds, J.; Hall, S.; Jiang, S. Quantitative detection of tetrodotoxin (TTX) by a surface plasmon resonance (SPR) sensor. *Sens. Actuators B Chem.* **2008**, *130*, 120-128.
